# A post-mortem study of respiratory disease in small mustelids in south-west England

**DOI:** 10.1186/s12917-016-0693-9

**Published:** 2016-04-06

**Authors:** Victor R. Simpson, Alexandra J. Tomlinson, Karen Stevenson, Joyce A. McLuckie, Julio Benavides, Mark P. Dagleish

**Affiliations:** Wildlife Veterinary Investigation Centre, Chacewater, Truro, Cornwall TR4 8 PB UK; The Paddock, Newtown, Longnor, Buxton, Derbyshire SK17 0NE UK; Moredun Research Institute, Pentlands Science Park, Bush Loan, Edinburgh, Midlothian EH26 0PZ UK; Instituto de Ganaderia de Montaña (CSIC-ULE), Grulleros, León, 24346 Spain

**Keywords:** Mustelid, Respiratory disease, *Angiostrongylus*, *Skrjabingylus*, Pyothorax, Granuloma, Adiaspore, *Emmonsia*, *Mycobacterium*

## Abstract

**Background:**

Stoat (*Mustela erminea*) and weasel (*Mustela nivalis*) populations in south-west England are declining whilst polecats (*Mustela putorius*), absent for over a century, are increasing. Little is known about the health status of these species nationally. This study aimed at investigating respiratory disease in specimens found dead in south-west England.

**Results:**

Trauma caused by road traffic, predator attack or being trapped was the predominant cause of death in 42 stoats, 31 weasels and 20 polecats; most were in good physical condition. *Skrjabingylus nasicola* was present in all species (weasels 37 %, polecats 39 %, stoats 41 %) and infected animals showed no evidence of loss of body condition. Even in carcases stored frozen L_1_ larvae were frequently alive and highly motile. *Angiostrongylus vasorum* infection was diagnosed in two stoats and one weasel: in stoats infections were patent and the lung lesions were likely of clinical significance. These are believed to be the first records of *A. vasorum* in small mustelids. Pleuritis and pyothorax was seen in two polecats, in one case due to a migrating grass awn. Histological examination of lungs showed granulomata in stoats (38 %), weasels (52 %) and polecats (50 %). Spherules consistent with *Emmonsia* spp. adiaspores were present in the granulomata of stoats (60 %), weasels (36 %) and polecats (29 %). Adiaspore diameter in all three species was similar (means: stoats 39 μm, weasels 30 μm, polecats 36 μm); these are markedly smaller than that normally recorded for *E. crescens.* Although they lie within the accepted range for spores of *Emmonsia parva* this arid-zone species is not found in Britain, thus raising a question over the identity of the fungus. Cases showing numerous granulomata but few or no adiaspores were Ziehl-Neelsen-stain negative for acid-fast bacilli and IHC negative for *Mycobacterium* spp*.* However, in some cases PCR analyses revealed mycobacteria, including *Mycobacterium kumamotonense* and *Mycobacterium avium* Complex. One stoat had numerous unidentified small organisms present centrally within granulomata.

**Conclusions:**

Stoats, weasels and polecats in south-west England share several respiratory diseases, often of high prevalence, but the pathology would appear insufficient to impact on the health status of the populations and other ultimate causes of death should be investigated when examining these species.

## Background

Stoats (*Mustela erminea*) and weasels (*Mustela nivalis*) are common and widespread in Britain but are understudied and poorly understood [1]. Although at a national level the populations of both species are thought to be either stable or declining [2, 3], in south-west England the evidence of declines is clearer, particularly in weasels [4]. The reasons for the declines are unknown but possibly include reduced prey availability as a result of changes in agricultural practices, secondary poisoning by rodenticides and, in the case of stoats, increased predation by an increasing fox population [2, 5, 6]. Formerly widespread, the polecat (*Mustela putorius*) was persecuted almost to the point of extinction during the late 1800s, with only a small number surviving along the English Welsh border. During the latter half of the 1900s this population started to recover and in recent decades polecats have recolonised most of Wales and much of central England [7, 8]. Since 2010, increasing numbers of polecats have been recorded in Somerset, Devon and Cornwall (Simpson, V., Couper, D. and Williams, J. unpublished data).

Apart from a study of stoats by McDonald and co-workers in 2001 [3] there have been no surveys to investigate the health status of stoats, weasels and polecats in Britain. However, a number of disease conditions have been identified in British small mustelids, mostly in studies targeting a specific organism. *Mycobacterium bovis* infection was recorded in a small number of stoats [9], *Mycobacterium paratuberculosis* in stoats [10] and *Neospora caninum* infection in polecats [11]. Canine distemper occurs worldwide [12] but whilst the disease has been well documented in mustelids in Europe [13] the only recorded cases in Britain were in captive stoats and weasels [14]. Evidence of Aleutian disease has been found in various mustelids in mainland Europe [15, 16] but although a high antibody prevalence was recorded in feral American mink (*Mustela vison*) in south-east England [17], the infection has not been proven in other British small mustelids. Two conditions affecting mustelids that are well documented in Britain, as in many other countries, are adiaspiromycosis due to *Emmonsia* species fungi [18, 19] and upper respiratory tract infection by the nasal nematode *Skrjabingylus nasicola* [20, 21].

McDonald and co-workers [3] concluded that the stoats that they examined from eastern England were remarkably healthy apart from respiratory disease of undetermined aetiology. In the absence of other surveys to determine the health status of stoats, weasels or polecats in Britain the pathology and epidemiology of any diseases that may affect them are largely unknown [22]. The purpose of the present study was to examine further the causes of respiratory disease in these three species of small mustelids in south-west England and to consider their possible impact on the health of the populations.

## Methods

This was an opportunistic study during 1999 to 2014 in which small mustelids found dead in south-west England were collected by members of the public and conservation bodies and submitted for post-mortem examination. The first polecats were submitted in 2011 when several were trapped on an estate in Somerset as part of its normal pest control programme; thereafter most were road traffic casualties submitted by members of the public. Polecats whose pelage was not consistent with that of a true polecat [23] were not included in the study. Carcases submitted in a fresh state were normally examined on the day of receipt or, failing that, within 24 h. Carcases submitted frozen were kept at -20 °C until they could conveniently be thawed and examined. Each specimen was given a unique identification number, weighed, and sexed prior to post-mortem examination. Animals were aged as adult, subadult or immature based on their size, dental wear and gonadal development. Body condition was subjectively assessed, based separately on fat deposits and muscle condition. In each case, fat and muscle condition were assigned to one of three categories: good; moderate; and poor/nil. In some instances it was not possible to reliably assess condition, due to autolysis and/or trauma. In freshly dead specimens with lesions suggestive of a bacterial infection tissue samples or swabs were submitted to Animal Health and Veterinary Laboratories Agency (AHVLA), Truro, for bacteriological examination. Irrespective of whether gross pathological lesions were seen, samples of lung and heart were routinely placed in 10 % buffered formal saline, processed routinely through graded alcohols, embedded in paraffin wax, sectioned at 5 μm, stained by haematoxylin and eosin and, in selected cases, by per-iodic acid Schiff (PAS), Giemsa, Gram and Ziehl-Neelsen (ZN). Granulomata and spores were measured, where possible, using an eye-piece micrometer calibrated against a stage micrometer. Mean granulomata and spore diameters for each mustelid species were derived by pooling the measurements of each granuloma or spore from each individual specimen, using a maximum of 10 values per specimen. The same procedure was deployed to examine histological sections of ten Eurasian otters’ (*Lutra lutra*) lungs held in one author’s (VRS) archive from previous studies [19, 24, 25]. Nasal passages were routinely irrigated via the nasopharynx with a small quantity of tap water and a drop of recovered fluid placed on a microscope slide and examined by direct microscopy for parasites. In selected cases scrapes of tracheal mucus and wet impressions from cut surface of lung were also examined by direct microscopy. A variation on the Baermann technique was used to examine the lungs of a stoat for first stage larvae of *Angiostrongylus vasorum*. Briefly, representative samples of each lobe were pooled, macerated in water and enclosed in a piece of cotton gauze. This was then held in the neck of a conical centrifuge tube and tap water slowly added until no air space remained in the tube. After standing overnight, the gauze and lung tissue was removed, the bulk of the water pipetted off and a sample from the bottom of the tube transferred to a microscope slide for examination by direct light microscopy.

To investigate possible infection by *Mycobacterium* species in five selected mustelids with granulomata, various PCRs were performed. DNA was extracted from 20 μm sections of formalin fixed, paraffin embedded tissue. Briefly, paraffin was removed by the addition of xylene, the tissue pelleted and then washed twice with ethanol. The tissue was lysed using 0.1 mm silica beads (Lysing Matrix B, MP Biomedicals) in AQL tissue lysing buffer (Qiagen DNeasy Kit) in a FastPrep™ FP120 (Thermo Savant) for three cycles of 20 s at 6 m/s with cooling on ice in between. Beads and debris were pelleted in a microfuge and the supernatant transferred to a fresh microcentrifuge tube. After overnight treatment with Proteinase K, DNA was extracted using the spin column procedure according to Qiagen DNeasy Kit instructions. A pan mycobacteria PCR targeting the *hsp65* gene was performed as described by Telenti et al. [26]. Sections of ovine ileum with multibacillary paratuberculosis were used as extraction and positive controls and sterile distilled water as a PCR negative control. Amplified DNA was extracted from a 2 % agarose gel using a QIAquick PCR purification kit (Qiagen) and then sequenced (MWG BioTech) to identify the *Mycobacterium* species present. To differentiate further between members of the *Mycobacterium avium* Complex, PCRs for IS*900* specific for *Mycobacterium avium* subsp. *paratuberculosis* [27] and IS*901*specific for *Mycobacterium avium* subspecies *silvaticum* and subspecies *avium* [28] were performed. Using material from the same five animals, immunohistochemistry specific for *Mycobacterium* spp. was performed on 3.5 μm thick tissue sections placed on coated microscope slides (FLEX IHC microscope slides, Dako, Agilent technologies, Glostrup, Denmark). Sections were dewaxed in xylene, rehydrated in graded alcohols and then endogenous peroxidase was blocked by immersion in 3 % H_2_O_2_ in methanol solution (v/v) for 30 min in darkness at room temperature. Slides were subsequently rinsed twice in PBS (pH 7.4) and incubated with rabbit anti-*Mycobacterium avium* sbsp *paratuberculosis* polyclonal antiserum (which is known to detect several diverse species of mycobacteria; Julio Benavides, Mark Dagleish personal observations), diluted 1/9000 in PBS [29] overnight at 4 °C in a humidified chamber. After extensive washing in PBS, sections were incubated with a commercial visualisation kit (EnVision®+/HRP solution, Dako, Agilent technologies, Glostrup, Denmark) as per manufacturer’s instruction for 40 min at room temperature. After washing in PBS, antibody localization was visualised with AEC substrate-chromogen (AEC plus, Dako, Agilent technologies, Glostrup, Denmark). Sections were counterstained with Mayer’s haematoxylin for 10 s prior to mounting. Appropriate species- and isotype- matched immunoglobulins were used as negative controls.

To determine if the presence of *S. nasicola,* or the presence of pulmonary granulomata were associated with body condition, Fisher’s exact test was performed for each of the following: fat condition and the presence of *S. nasicola*; fat condition and the presence of pulmonary granulomata; muscle condition and the presence of *S. nasicola*; and finally muscle condition and the presence of pulmonary granulomata. Significance was assigned for *p* < 0.05. Analyses were run in R version 3.1.0.

## Results

Forty two stoats, 31 weasels and 20 polecats were examined. The gross pathology in the majority of cases was consistent with death due to trauma, mostly caused by road traffic, attack by predators or being trapped (Table 1). The majority of animals were in good body condition whether assessed by their fat deposits or muscle condition. Fat deposits were assessed in 88 cases and were good in 56 % (*n* = 49), moderate in 35 % (*n* = 31) and nil/poor in only 9 % (*n* = 8). Muscle condition was assessed in 86 cases and was good in 94 % (*n* = 81), moderate in 5 % (*n* = 4) and nil/poor in just 1 % (*n* = 1).

**Table 1:** Mortality due to trauma: the number of each species submitted, the number and proportion killed by road traffic, predation or other forms of trauma

Species	*n*=	Road traffic (%)	Predation (%)	Other trauma (%)	Total trauma (%)
Stoat	42	31 (74)	8 (19)	2 (5)	41 (98)
Weasel	31	7 (23)	20 (65)	3 (10)	30 (97)
Polecat	20	12 (60)	0	7 (35)^a^	19 (95)

^a^6 (30 %) trapped

In stoats and weasels killed by predators the pattern of the bite wounds was similar, with the majority of punctures across the caudal neck and thorax. In stoats the size and spacing of the bite wounds was mostly consistent with attack by foxes, or possibly similar size dogs, but in weasels the wounds, and often the history, was consistent with them being killed by domestic cats. No polecats were killed by predators and most of the non-road traffic deaths were caused by traps. No details of the traps were available but the pattern of bruising and haemorrhage to the skin over the neck and thorax suggested they were Fenn-type spring traps.

### Upper respiratory tract

**Fig. 1:**
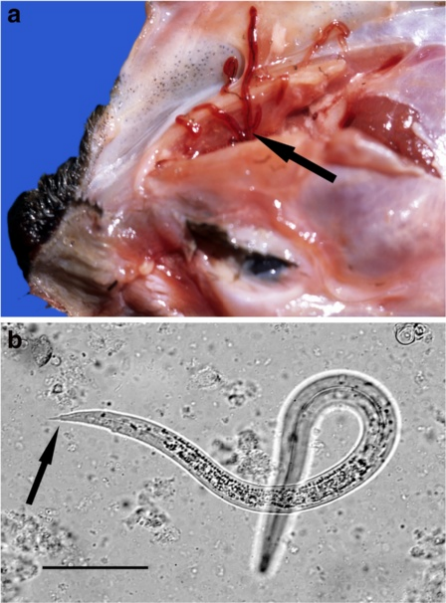
*Skrjabingylus nasicola*. **a** Reflection of the skin over the frontal area of the crushed skull of a stoat killed by road traffic revealed adult *Skrjabingylus nasicola* in the nasal passages. **b** *S. nasicola* first stage larva flushed from the nasal passages of a stoat. Note the stepped but pointed tail tip that is characteristic of the species (*arrow*). Bar = 50 μm

Microscopic examination of fluid flushed from the nasal passages showed first stage larvae of *Skrjabingylus nasicola* in all three mustelid species with a prevalence of 37 % in weasels, 39 % in polecats and 41 % in stoats (Table 2). Remarkably, on defrosting the carcases of five stoats and three weasels that had been frozen, ranging from 10 days to 13 months, first stage larvae were still alive, and in some cases highly motile. Weakly viable and non-viable larvae were seen in two of the frozen polecats. Confirmatory checks in selected cases demonstrated adult nematodes in the nasal passages, especially the frontal and maxillary sinuses (Fig. 1). Examination was not possible in every case due to extensive trauma; this was most severe in those animals killed by road traffic.

**Table 2:** The prevalence of infection with *Skrjabingylus nasicola* for stoats, weasels and polecats

Species	Number positive	Number examined	% positive (95 % CI)
Stoat	14	34	41 (26–58)
Weasel	10	27	37 (22–56)
Polecat	7	18	39 (20–61)

Bone deformity due to the presence of *S. nasicola* was not common and in most cases the lesion was considered to be of little clinical significance. Fisher’s exact test confirmed that there was no statistically significant association between the presence of *S. nasicola* and body condition based on either fat deposits or muscle condition: in each analysis *p*>> 0.05.

**Fig. 2:**
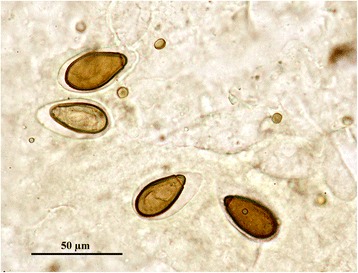
Fluke eggs recovered from a polecat. These eggs were present in fluid flushed from the nasal passages of a polecat in Cornwall. There was no associated gross pathology in this or in a second case where eggs were seen

Fluid flushed from the nasal passages of two polecats contained thick walled, operculate, trematode eggs (Fig. 2). They had a mean length of 31.8 μm (range 30.6 - 32.9 μm, *n* = 4). The head of each polecat was cut through the mid sagittal plane but no adult trematodes were seen in the nasal cavities. A single, small, immature fluke was seen in nasal fluid from a third polecat. The first two animals were from the same area of east Cornwall and the third was from south–east Cornwall; all were sub-adults killed by road traffic. None of the animals had skull lesions consistent with parasite infection.

### Lower respiratory tract and heart

**Fig. 3:**
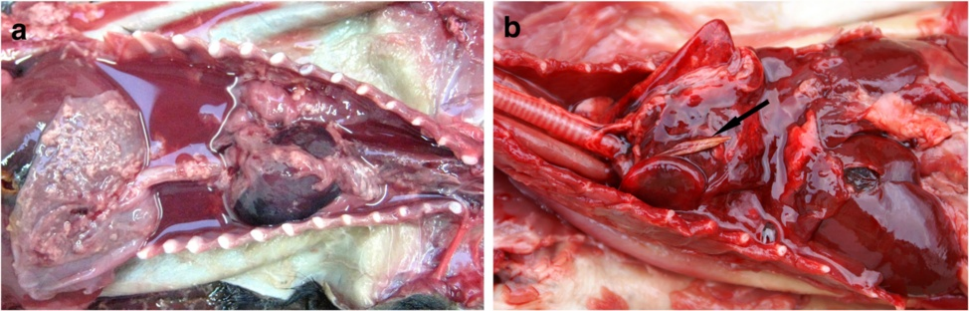
Pleuritis in polecats. **a** This animal shows advanced pyothorax and haemopericardium. Numerous Gram positive cocci and a small number of Gram negative bacilli were present in the purulent fluid but the source of infection was not established. **b** Pleuritis caused by a grass awn (*arrow*)

In most animals the predominant lesions within the thorax were caused by trauma and specific abnormalities were uncommon. Two cases of pleuritis and pyothorax were seen in polecats from Somerset. The first (Fig. 3a) was an immature male with emerging permanent teeth. It was in poor muscular condition and had no subcutaneous fat deposits. The thoracic cavity contained a large amount of dark, reddish-brown purulent fluid and amorphous floccular material; the parietal and visceral pleura were greatly thickened and covered in pinkish-cream coloured amorphous deposits. There were extensive pleural adhesions on the right side and the lungs were partly collapsed with areas of patchy congestion and consolidation. The pericardial cavity was filled with dark red fluid consistent with haemopericardium. Gram stained impression smears from the pleura showed large numbers of Gram positive coccal bacteria, often in chains, and small numbers of Gram negative bacilli. No likely source of infection was evident. It was suspected that the animal might have been trapped, but there was no clear evidence and it had been submitted without any history.

The second polecat with pleuritis (Fig. 3b) was a subadult female in good body condition. The right side of the thoracic cavity contained a large amount of dark reddish-brown fluid, the lobes of the right lung were collapsed, distorted and consolidated and there were extensive adhesions to the parietal pleura, pericardium and mediastinum. Within the adhesions and fluid was an approximately 25 mm long grass awn. The left lung showed mild, patchy congestion. Gram stained impression smears of the pleura showed masses of cellular debris but no organisms. There were subcutaneous lesions over the head and neck that were consistent with the animal having been killed in a spring trap. The stomach contained the undigested remains of a small mammal showing that, despite the pleuritic lesions, the animal had been hunting shortly before it was killed.

**Fig. 4:**
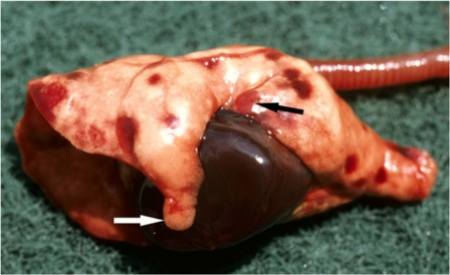
Pasteurella septicaemia in a stoat. The lungs show multiple irregular areas of haemorrhage; there is also consolidation (*black arrow*) and emphysema along the ventral border of the cardiac lobe (*white arrow*). *Pasteurella multocida* was isolated in pure culture from the lungs and from the heart

The single stoat that did not die as a direct result of trauma had multiple haemorrhagic lesions in both lungs, foci of consolidation and emphysema along the ventral margins (Fig. 4). *Pasteurella multocida* was isolated in pure culture from lung and heart blood.

**Fig. 5:**
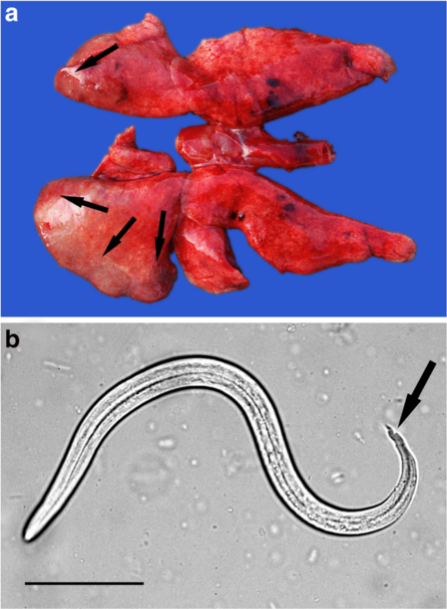
Angiostrongylosis in a stoat. **a** The lungs show areas of consolidation and swelling (*arrows*) along the ventral margins of the diaphragmatic lobes caused by *Angiostrongylus vasorum* infection. The areas of haemorrhage in the cranial lobes were a result of trauma caused by road traffic. **b** One of many first stage larva of *A. vasorum* seen in a wet impression taken from the cut surface of the lung. Note the wavy, double notched tail-tip which is characteristic of *A. vasorum* (*arrow*). Bar = 50 μm

**Fig. 6:**
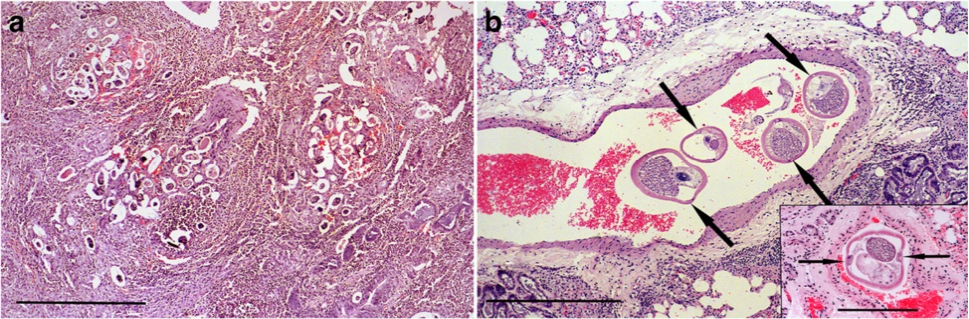
Histological lesions of angiostrongylosis in a stoat and a weasel. **a** Section of lung from the stoat shown in Fig. 5 showing clusters of *A. vasorum* eggs in various stages of development surrounded by haemorrhage, granulomatous reaction and fibrosis. Haematoxylin and eosin stain, bar = 500 μm **b**. Transverse section of four adult *A. vasorum* (*arrows*) in the pulmonary artery of the same stoat. Haematoxylin and eosin stain, bar = 500 μm. Inset: Transverse section of a nematode in the pulmonary artery of a weasel. Its location within the artery and the clearly visible lateral chords (*arrows*) are consistent with *A. vasorum.* Haematoxylin and eosin stain, bar = 200 μm

An adult male stoat killed by road traffic had well defined, swollen, purplish-brown areas of consolidation along the ventral and caudal margins of the diaphragmatic lobes and markedly enlarged bronchial and mediastinal lymph nodes (Fig. 5a). Examination of the heart revealed a red, adult, female nematode measuring 18 mm in the right ventricle. Macerated samples of lung were examined by modified Baermann technique and showed numerous first stage metastrongyle-type larvae, the tail morphology of which was consistent with that of *Angiostrongylus vasorum* (Fig. 5b). The adult nematode was submitted to Bristol university where sequencing of the 18 s rDNA ITS2 gene confirmed it to be *A. vasorum*. Histological examination of the stoat’s lung showed widespread areas of consolidation, multiple foci of parasite eggs in various stages of development surrounded by a granulomatous reaction and adult nematodes in the pulmonary artery (Fig. 6).

Angiostrongylosis was also diagnosed in a second stoat and a weasel. The stoat was an adult male and had been killed by road traffic. It had a well-circumscribed, firm, consolidated swelling in the caudal half of the left diaphragmatic lobe and the bronchial and mediastinal lymph nodes were enlarged. Microscopic examination of wet impressions from the cut surface of the lung revealed many first stage larvae of *A. vasorum*. Bacteriological cultures of lung and lymph nodes for *Mycobacterium* spp. proved negative. Histological examination of lung showed little normal pulmonary parenchyma but multiple, coalescing, granulomata enclosing groups of embryonated eggs and nematode larvae. The weasel was an adult male killed by road traffic and in good body condition. Extensive trauma prevented detailed gross examination of the lungs but histological examination showed adult nematodes with the morphological features of *Angiostrongylus* species within branches of the pulmonary artery (Fig. 6 inset).

**Fig. 7:**
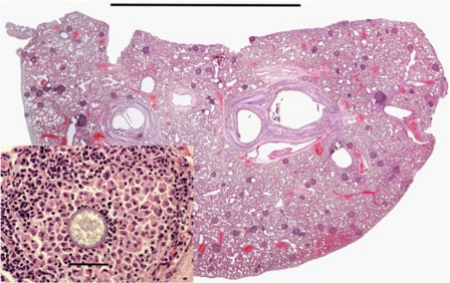
Pulmonary granulomatosis in a polecat. Low power magnification of a section through a polecat lung showing numerous granulomata (*purple, roughly circular lesions*). The granulomata were not apparent during gross pathological examination. H & E stain, bar = 1 cm. Inset: Higher magnification view of one granuloma in the section which shows a central body consistent with an adiaspore. H & E stain, bar = 50 μm

The lungs of 61 of the 93 (66 %) mustelids submitted were suitable for histological examination and, excluding any changes due to trauma, lesions were observed in 40 cases (66 %). In all three species small granulomata of similar size and structure were the predominant lesion. The granulomata ranged from one or two in a section of lung to numerous (Fig. 7). One lobe, or part of a lobe, was often more severely affected than the rest. As for *S. nasicola*, Fisher’s exact test confirmed that there was no statistically significant association between the presence of pulmonary granulomata and body condition based on either fat deposits or muscle condition: in each analysis *p*>> 0.05. Foci of metaplastic bone and/or osteoid were also common. Other lesions seen included diffuse areas of inflammatory cell infiltration, granulomata with cholesterol-type clefts, thrombosed blood vessels and, in one case, unidentified schizont-like bodies.

**Fig. 8:**
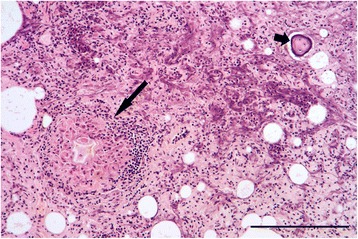
Granuloma in the lung of a stoat. The granuloma (*large arrow*) is composed of a central amorphous mass surrounded by macrophages, a thin layer of epithelioid macrophages and then an outer layer composed predominantly of lymphocytes and fibroblasts. Also present is a focus of metaplastic bone (*short arrow*). H & E stain, bar = 200 μm

The structure of the granulomata varied. There was typically a central zone composed of large macrophages in the midst of which there was often amorphous acellular debris. In some cases this was bounded by concentric layers of epithelioid macrophages and occasional multinucleate giant cells but in others it merged directly to an outer zone composed mostly of lymphocytes with smaller numbers of neutrophils, eosinophils and fibroblasts (Fig. 8).

**Fig. 9:**
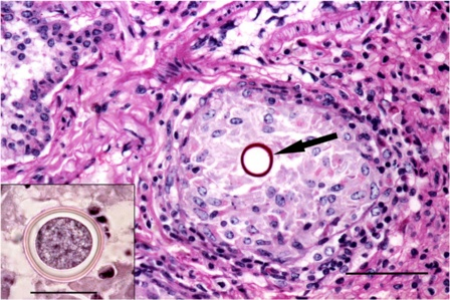
Examples of spores present in granulomata. Section of weasel lung showing a granuloma surrounding a central spore with a PAS positive cell wall (*arrow*); in this example the spore appears to be devoid of content. The spore is surrounded by a loose aggregation of macrophages and an outer layer of mostly lymphocytes and fibroblasts. Per-iodic Schiff stain, bar = 50 μm. Inset: High magnification view of a spore within a granuloma of a stoat showing the double-wall and the inner body. H & E stain, bar = 25 μm

Where large numbers of granulomata were present they sometimes formed a confluent, fibrosed, highly cellular mass that effaced the normal parenchyma. In some, but not all specimens, the central zone of one or more granulomata contained a roughly spherical, spore-like body or spherule consistent with an adiaspore of *Emmonsia* sp. (Table 3). Apparently empty adiaspores were common but others contained either a finely partitioned, roughly spherical body or a condensed mass of basophilic debris. When cut in mid plane, the spore wall was double layered and approximately 1–2 μm thick; it stained deep red by PAS (Fig. 9) or pink by haematoxylin and eosin (Fig. 9 inset). Fragmented or disrupted spores surrounded by inflammatory cells were also commonly present.

**Table 3:** Summary of histological examination of lungs, showing the number and proportion which contained granulomata and metaplastic foci of bone or osteoid and the number and proportion of the granuloma cases that had adiaspores

Species	Histology *n*=	Granuloma + ve (%)	Adiaspore + ve (%)	Bone/osteoid + ve (%)
Stoat	26	10 (38)	6/10 (60)	6/26 (23)
Weasel	21	11 (52)	4/11 (36)	3/21 (14)
Polecat	14	7 (50)	2/7 (29)	8/14 (57)

**Fig. 10:**
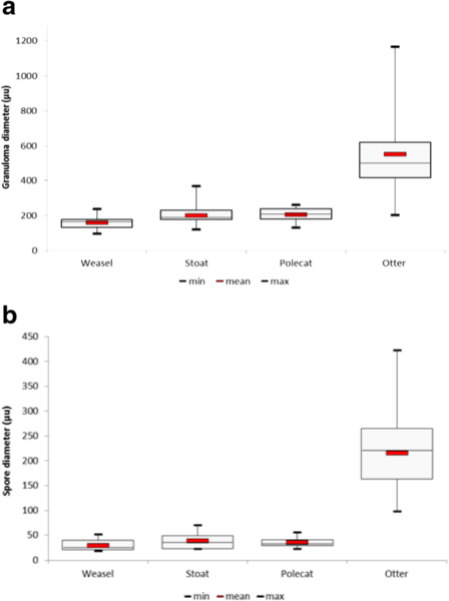
Box and whisker plots comparing granulomata and spore size between species. **a** Granulomata in stoats, weasels and polecats were markedly smaller than those in Eurasian otters. **b** With mean diameters of less than 40 μm, the spores in the small mustelids were consistently less than those in Eurasian otters (mean 216 μm.) Mean values shown by red bars

The proportion of granulomata in a section that contained spores was often low, for example, in one stoat only three spores were visible in 44 granulomata. In cases where granulomata were present but no spore was seen initially, sequential sections sometimes revealed the presence of a spore. However, in others cases repeated sectioning failed to show the presence of any spores. The mean diameters for both granulomata and spores, respectively, observed in stoats (202 μm; 39 μm), weasels (161 μm; 30 μm) and polecats (205 μm; 36 μm) were markedly smaller than those in archived sections from otters (550 μm; 216 μm) (Fig. 10a and b).

**Fig. 11:**
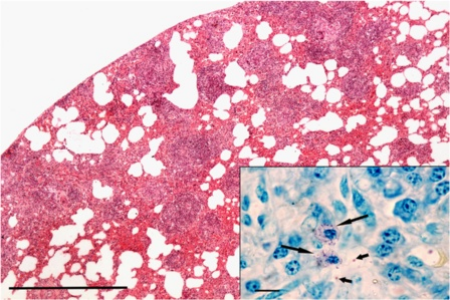
Granulomata in stoat lung due to unidentified microorganisms. Numerous granulomata are present but no adiaspores were seen in this case. H & E stain, bar = 500 μm. Inset: Unidentified organisms in the centre of a granuloma. Most are within the cytoplasm of macrophages (*large arrows*) but some are free in intercellular spaces (*short arrows*.) ZN stain, bar = 10 μm

In ten cases where granulomata were present, sometimes in large numbers, no adiaspores were seen despite examination of repeat sections. Further sections in each case were stained by ZN and all proved negative for acid-fast bacilli. However, five of these cases with large numbers of granulomata were further investigated by PCR and amplification of the *hsp65* gene obtained. For four of the cases, two stoats and two weasels, sufficient DNA was amplified to permit sequencing of the product and identification of the *Mycobacterium* species present. *Mycobacterium kumamotonense* (related to *Mycobacterium terrae* Complex) was identified in one stoat and one weasel. *Mycobacterium avium* Complex was identified in another weasel and stoat; the weasel was positive for *Mycobacterium avium* subsp. *paratuberculosis* and the stoat was positive for both *Mycobacterium avium* subsp. *paratuberculosis* and *Mycobacterium avium* subsp. *avium/Mycobacterium avium* subsp. *silvaticum*. The fifth case was a stoat which had shown enlarged retropharyngeal and prescapular lymph nodes at the time of post-mortem examination: samples of lung and lymph nodes had been submitted for bacteriological examination for *Mycobacterium* spp. but proved negative. Histological examination had shown numerous granulomata containing unidentified micro-organisms. Most were clustered within the cytoplasm of macrophages in the central zone of the granulomata but individual organisms were also seen in adjacent intercellular spaces. Within macrophages they were mostly ovoid or slightly elongate but some free organisms were ovoid or slender with what appeared to be a terminal nucleus. The cytoplasm stained pale pink by ZN and the nucleus mauve or purple (Fig. 11). Further investigation by PCR resulted in weak amplification of the *hsp65* gene suggesting a mycobacteria infection but it was not possible to obtain sufficient DNA to confirm this. Further identification of this organism was beyond the scope of this work. Examination by IHC of lung sections from these five cases showed no specific labelling of mycobacteria in any of the granulomata or other tissue found in these sections. However, in the process of carrying out the IHC up to seven sections per case were examined and between one or two adiaspores were seen in three animals, two weasels and a stoat. There was no evidence of spores in the stoat which had granulomata infected by unidentified organisms. All of these cases had previously proved negative for adiaspores.

## Discussion

Infection of stoats, weasels, polecats and other small mustelids by the metastrongyloid parasite *Skrjabingylus nasicola* has been well documented in Europe and elsewhere in the World [30, 31]. Although a range of gastropods act as intermediate hosts it is thought that these are rarely eaten by small mustelids and it is likely that they become infected by eating small mammals which act as paratenic hosts, notably wood mice (*Apodemus sylvaticus*), bank voles (*Clethrionomys* (*Myodes*) *glareolus*) and, in some areas, shrews (*Sorex* sp.) [32, 33]. In the final host, ingested larvae migrate to the central nervous system and travel via the subarachnoid space to the front of the brain. Here they follow the olfactory nerves through the cribriform plate to enter the nasal sinuses where they mature [34]. The irritation caused by the adult worms can result in erosion, remodelling and deformity of the frontal bones.

The lesions to the frontal area of the skull are often considered pathognomonic for *S. nasicola* infection and have been widely used in studies to determine the prevalence and distribution of the parasite [30, 35, 36]. However, infection with the trematode *Troglotrema acutum* can also result in similar skull lesions that cannot be reliably distinguished from those caused by *S. nasicola* [37, 38]. Although *T. acutum* is commonly found in small mustelids in Continental Europe, especially in polecats [37, 38], there appear to be no confirmed records of it occurring in the UK ([39], Harris, E. personal communication). Trematode eggs that morphologically resembled those of *T. acutum* were recovered from the nasal passages of two polecats in the present study but they were significantly smaller than those typically recorded for *T. acutum* [37, 38]. It appears that no trematode species has been recorded in the upper respiratory system of polecats in the UK (Bray, R and Harris, E personal communication) and further studies are required to collect and identify the trematodes demonstrated in this study.

In the present study, many specimens, especially those killed by road traffic, had suffered severe damage to the head which often precluded meaningful examination for skull lesions. However, the technique of irrigating the naso-pharynx, nasal passages and turbinates with a small amount of water and then looking for first stage larvae in recovered fluid not only overcame the problem caused by trauma but also identified early stage infection where bone lesions were not apparent. The fact that the larvae were normally highly motile, even in carcases that had been frozen, made them easy to locate when only small numbers were present in a sample. The prevalence figures in the present study are comparable with those reported previously in England and elsewhere in Europe [30] and, as has been observed previously [20], infection with *S. nasicola* did not appear to adversely affect an animal’s body condition.

*Angiostrongylus vasorum* is possibly the most significant parasite of dogs in Britain. Its common epithet ‘French Heartworm’ alludes to the fact that the first description and most of the pioneering work on the parasite was performed in Toulouse in south-west France [40, 41]. Since then, particularly during the mid to late 1900s, the parasite has extended its range remarkably and is now found on most continents. The first reports of autochthonous infection in domestic dogs in Britain were from Cornwall in the early 1980s [42, 43] and the first cases in foxes in 1996 [44]. During the next 30 or so years the parasite spread steadily northwards throughout most of England and Wales [45] and its range now extends to all but the northern part of Scotland [46, 47]. The principal means of spread in Britain has almost certainly been due to the translocation of infected dogs. However, foxes also play a role in the epidemiology of the disease by maintaining local reservoirs of infection.

Apart from foxes there has, until now, been no evidence of *A. vasorum* infection in other free-living species of wildlife in Britain. Larvae identified as those of *A. vasorum* were seen in the lungs of a single Eurasian otter (*Lutra lutra*) in Denmark [48] but post-mortem examinations on 700 otters in Britain, many of which came from known endemic areas of infection, all proved negative for the parasite [49]. Kirk and co-workers [46], citing Guilhon [50], suggested that Eurasian otters could act as an alternative final host but this was in error as Guilhon [50] made no mention of infection in otters. Although *A. vasorum* has been reported in badgers (*Meles meles*) in Spain and Italy [51, 52], large numbers of badgers have been examined in Britain in connection with the control of bovine tuberculosis and, whilst occasional cases of *Aelurostrongylus falciformis* have been seen ([53], A. Barlow, pers. comm.), there have been no reports of *A. vasorum* infection.

A literature study of diseases of stoats and closely related mustelids found no record of infection with *A. vasorum* [31] and a post-mortem study of stoats from various locations in eastern England by McDonald and co-workers also proved negative for the parasite [3]. The latter study did detect nematodes in the lungs of five stoats but these were not identified; however, a figure in the article [3] showed adult females within which were numerous larval forms in various stages of development. The nematodes were in the pulmonary parenchyma but not in the pulmonary artery. These features, together with the fact that larvae but no eggs were present in the parenchyma, suggest that the parasite was probably *Aelurostrongylus falciformis*. No similar parasites were seen in any of the mustelids in the present study but there was unequivocal evidence of *A. vasorum* infection in two stoats. Confirmation of the identity of the parasite in the weasel was lacking, but their location within the pulmonary artery, the characteristic morphology with lateral chords, and the fact that no other *Angiostrongylus* species is known to exist in south-west England, all support the presumptive identification of *A. vasorum*. The clinical significance of the pulmonary lesions in the stoats in this study is uncertain but they may well have been sufficient to impair the animal’s hunting ability.

Like *S. nasicola*, *A. vasorum* is a metastrongyloid parasite with a life cycle that depends on a gastropod intermediate host. Domestic dogs are thought to become infected by eating slugs whilst foxes, especially cubs, are known to eat various molluscs. Foxes will also eat frogs, which are considered to be a paratenic host for *A. vasorum* [54]*.* However, as stoats and weasels are thought to rarely eat any of these prey items the question arises whether, as with *S. nasicola*, another paratenic host exists for *A. vasorum*.

Pleuritis associated with pyothorax was seen in two polecats but not in any of the stoats or weasels. One case was due to a grass awn which had most likely been inhaled, migrated through the lung parenchyma and penetrated the pleura. This is a well-recognised cause of pyothorax in dogs, especially those used for hunting or living in rural environments, and may also occur in cats [55]. The cause of pyothorax in the other case was not established but it may also have been due to a grass awn as they can be nearly impossible to find where there is copious pleural exudate [55]. Pyothorax occurs in Eurasian otters where it is typically associated with either septic bite wounds caused by intraspecific aggression or septic tooth lesions [25] but the polecat was a young animal with emerging, apparently healthy, permanent teeth and no visible bite wounds. One stoat died as a result of a *Pasteurella multocida* septicaemia. This is a well-recognised cause of mortality in small mammals, bats and small birds submitted to wildlife hospitals, especially where there is a history of them having been bitten by domestic cats. External lesions are often minimal but septicaemia and death typically occurs around 3 days after being bitten [56, 57].

The predominant histological feature in the lungs of all three species was the presence of small granulomata, some of which contained a central spore. The morphology and staining of the spores was consistent with the adiaspores of *Emmonsia* (*Chrysosporium*) species. These fungi have a saprophytic phase where mycelia growing on decaying plant material in soil produce sporangia that release aleuriospores. If these spores are inhaled by a mammal their small size (ca 2–4 μm) enables them to enter alveolar spaces; here they produce a double layered wall and increase markedly in volume to become adiaspores. The life cycle cannot progress until the mammalian host dies and the adiaspores are released into the environment. The genus *Emmonsia* contains two species that are capable of producing adiaspores, *E. crescens*, which is the predominant species in Europe and produces multinucleate adiaspores of 200–700 μm in diameter, and *E. parva* which is found in hotter, dryer climates and produces mononuclear adiaspores that typically only grow to around 20–40 μm [58, 59].

Adiaspiromycosis has been recorded in many species of small mammals throughout the World, especially members of the Family Mustelidae [18, 19, 60–64]. A notable feature in all three species in the present study was the consistently small size of the adiaspores, with none greater than 70.5 μm and mean values for each mustelid species of less than 40 μm. This is markedly smaller than those typically reported in otters where they often exceed 200 μm [18, 19, 65] and strikingly less than the mean value of 216 μm for the ten otters examined in this study (Fig. 10b). Granulomata due to *Emmonsia* infection are readily seen during gross pathological examination of otters [19] but none were observed in the small mustelids in this study. These results raise a question over the identity of the species infecting the stoats, weasels and polecats, especially as *E. parva* is not known to exist in south west England. In an earlier study of adiaspiromycosis in weasels in Finland the authors also commented on the fact that the adiaspores, measuring 28–64 μm, were significantly smaller than those in the rodents on which the weasels preyed and questioned whether weasels were better able to supress the growth of adiaspores [62]. If the adiaspores found in small mustelids are those of *E. crescens* and their small size is due to a host response, the quoting of size ranges in the literature as a means of differentiating between *E. crescens* and *E. parva* cannot be justified. Alternative explanations for the atypically small spores found in small mustelids are that they belong to neither of the two recognised species of *Emmonsia* capable of producing adiaspores or that they belong to another fungus species.

The high proportion of granulomata that did not contain an adiaspore in this study was in contrast to earlier studies in Eurasian otters where the majority of granulomata in a section of lung contained an adiaspore [19, 65]. A possible explanation for this would be the small size of the spores relative to that of the granulomata; more than 12 sections cut at 6 μm might be needed to locate a 40 μm spore within a 200 μm granuloma. A second reason might be that a higher proportion of spores are destroyed by the host response in the small mustelids than in otters. Ossification of scar tissue following the destruction of an adiaspore may be the reason for the foci of metaplastic bone and osteoid seen in many cases. The possibility of multiple infections in the small mustelids cannot be ruled out and some granulomata could be the consequence of another causal agent.

In an earlier histological study of stoats from East Anglia [3] the authors considered granulomatous inflammation to be the most significant pulmonary change. In some cases this had progressed to form distinct microgranulomata with central cores of macrophages, surrounded by a cuff of lymphoid cells. However, no causal agent was demonstrated. In those cases in the present study where numerous granulomata were present but no spores were seen consideration was given to the possibility that an infection other than adiaspiromycosis was present. Lesions of *Mycobacterium* species infection, especially *M. bovis*, can be confused with those of adiaspiromycosis [64, 66] and bovine tuberculosis is of particular concern in south west England where *M. bovis* is prevalent in the abundant badger population [67]. None of the ZN- stained sections in this study revealed evidence of acid-fast bacilli but mycobacteria can be sparse and difficult to locate in chronic lesions. However, investigation by PCR did not detect *M. bovis* but revealed the presence of *M. kumamotonense* and members of the *M.avium* Complex (*M.a. paratuberculosis* and *M.a. avium*/*silvaticum*). *M. kumamotonense* is related to the *M. terrae* Complex and has been misidentified as *M. tuberculosis* Complex by commercial probes. It is possible that the granulomata observed could be the result of infection by a number of different mycobacteria but further investigations would require laser capture microdissection and highly sensitive PCR techniques which were outside the scope of this study. The lack of labelling of *Mycobacterium* spp. by IHC was probably a sensitivity issue as PCR is an exceptionally more sensitive technique. Further screening of small mustelids in south west England for mycobacteria by bacteriological and molecular biological techniques would seem prudent.

## Conclusion

Respiratory disease is common in stoats, weasels and polecats in south-west England, all three species being particularly vulnerable to *Skrjabingylus nasicola* infection and pulmonary granulomatosis. There was no apparent loss of body condition associated with either disease but this was possibly influenced by the fact the animals had died prematurely due to trauma. The granulomata in approximately a third of cases, irrespective of species, contained fungal spores consistent with adiaspores of *Emmonsia* species but the spores were markedly smaller than the size normally quoted for *E. crescens* which raises doubts over their true identity. The detection of *Mycobacterium* species by PCR in several animals exhibiting large numbers of granulomata might be interpreted as a causal relationship. However, the failure to demonstrate mycobacteria by IHC using a polyclonal antibody makes it questionable as to whether the mycobacteria were actually responsible for the lesions. At present, it is only possible to say that the causes of granulomata in the three species of small mustelids in this study include a fungus, probably an *Emmonsia* species and an as yet unidentified organism. Pleuritis and pyothorax was seen in polecats but not in stoats or weasels. The demonstration of *Angiostrongylus vasorum* infections in stoats and weasels, which were proved to be patent in the stoats, means these species have the potential to play a role in the epidemiology of the disease. Overall, the pathology observed in the stoats and weasels in this study was not sufficiently different from that in polecats and would seem unlikely that the diseases observed could be responsible for stoat or weasel population declines in south-west England when polecats are increasing. However, in view of the expanding polecat population and the high prevalence of bovine tuberculosis in the region, it is recommended that further studies be carried out to identify and clarify the agent(s) responsible for the pulmonary granulomata.
